# Evolutionary and Comparative Expression Analyses of TCP Transcription Factor Gene Family in Land Plants

**DOI:** 10.3390/ijms20143591

**Published:** 2019-07-23

**Authors:** Ming-Ming Liu, Mang-Mang Wang, Jin Yang, Jing Wen, Peng-Cheng Guo, Yun-Wen Wu, Yun-Zhuo Ke, Peng-Feng Li, Jia-Na Li, Hai Du

**Affiliations:** 1 College of Agronomy and Biotechnology, Southwest University, Chongqing 400716, China; 2 Academy of Agricultural Sciences, Southwest University, Chongqing 400716, China

**Keywords:** TCP transcription factor, phylogenetic analysis, origin, evolution, interaction network, expression profile analysis

## Abstract

The plant-specific Teosinte-branched 1/Cycloidea/Proliferating (TCP) transcription factor genes are involved in plants’ development, hormonal pathways, and stress response but their evolutionary history is uncertain. The genome-wide analysis performed here for 47 plant species revealed 535 TCP candidates in terrestrial plants and none in aquatic plants, and that TCP family genes originated early in the history of land plants. Phylogenetic analysis divided the candidate genes into Classes I and II, and Class II was further divided into CYCLOIDEA (CYC) and CINCINNATA (CIN) clades; CYC is more recent and originated from CIN in angiosperms. Protein architecture, intron pattern, and sequence characteristics were conserved in each class or clade supporting this classification. The two classes significantly expanded through whole-genome duplication during evolution. Expression analysis revealed the conserved expression of TCP genes from lower to higher plants. The expression patterns of Class I and CIN genes in different stages of the same tissue revealed their function in plant development and their opposite effects in the same biological process. Interaction network analysis showed that TCP proteins tend to form protein complexes, and their interaction networks were conserved during evolution. These results contribute to further functional studies on TCP family genes.

## 1. Introduction

The plant-specific TCP transcription factor family genes are involved in multiple processes of plant growth and development, such as branching [1,2], leaf development [3], hormone pathways [4,5], seed germination [6], and circadian clock [7]. These transcription factor genes (TFs) are characterized by an N-terminal non-canonical beta helix-loop-helix (bHLH) domain known as the TCP domain. However, TCP TFs have little homology with bHLH TFs and bind to DNA elements distinct from those recognized by bHLH TFs [8]. The TCP proteins were initially identified and named after the first three family members characterized: TEOSINTE BRANCHED 1 (TB1) from maize (*Zea mays*) [9], CYCLOIDEA (CYC) from snapdragon (*Antirrhinum majus*) [10], and the PROLIFERATING CELL FACTORS 1 and 2 (PCF1 and PCF2) from rice (*Oryza sativa*) [8,11].

The TCP proteins are categorized into two classes, Class I and Class II, according to the sequence homology of their TCP domains [12]. Class I has a conserved four-amino-acid deletion in the TCP domain and some Class II genes display additional motifs such as a glutamic acid-cysteine-glutamic acid (ECE) stretch and/or an arginine-rich R-domain out of the TCP domain [11,12,13]. The members of Class II can be further subdivided into the CIN and CYC clades according to sequence differences within the TCP domains [14]. The CIN subclade is represented by *Antirrhinum* CINCINNATA (CIN) [15] and the CYC subclade is represented by CYC and TB1 [13]. Class II TCP proteins are known to regulate many plant processes, including development (e.g., leaf differentiation, axillary meristem development, shoot branching), hormone signaling, and defense, among others [2,5,9,10,16]. For example, *AtBRC1* in *Arabidopsis thaliana* [2], *OsFC1* in *O. sativa* [1], and *TgTB1* in *Tulipa gesneriana* [17] are involved in axillary bud development and branch control. In contrast, relatively less functional information is available for Class I TCP proteins/genes, although members of this class also participate in plant leaf development, growth, and proliferation [18,19]. For example, in *A. thaliana*, *AtTCP15* regulates internal replication and cytokinin and auxin responses during pistil development [4], *AtTCP14* and *AtTCP15* work together to regulate internode length [3] and seed germination [6], and *AtTCP23* participates in flowering time and plant development [20,21]. However, these two classes act antagonistically in regulating plant growth and development due to competition for their similar binding cis-regulatory sites [16,19].

Given the critical roles of TCP genes in diverse biological processes, TCP homologous genes have been analyzed in various plants, and analyses of this gene family have been performed across species genomes. For instance, six TCP genes were identified in *Physcomitrella patens* [12], 29 in maize [22], 66 in wheat (*Triticum aestivum*) [23], 24 in *A. thaliana* [24], 33 in *Populus euphratica* [25], and 74 in *Gossypium raimondii* [26]. These numbers evidence that the TCP gene family underwent a large expansion in higher plants. Although it has been reported that TCP proteins may have originated before land plant emergence [12], none of the studies conducted to date has shed light on the genome-wide expansion, evolution, structure, expression, and function of this gene family in land plants.

Thus, to improve knowledge on the evolutionary history of the TCP gene family in plants, we performed a genome-wide systematic identification of the TCP proteins in the major plant lineages (47 species), including chlorophytes, bryophytes, gymnosperms, eudicots, and monocots. This allowed for a comprehensive assessment of their origin, evolution, classification, and patterns of differentiation and proliferation in various phylogenetic groups. Our gene structure, evolution, and expression analyses demonstrated that this gene family emerged at the very beginning of land plants’ establishment and expanded fast during higher plants’ evolution. The TCP genes in the same class or clade generally showed highly conserved gene/protein structure and expression profile across land plants, suggesting their common origin and functional conservation. Interaction network prediction showed that this gene family tends to form protein complexes involved in diverse biological processes, such as phytohormone pathways, cell cycles, and environmental responses.

## 2. Results

### 2.1. TCP (Teosinte-Branched 1/Cycloidea/Proliferating) Proteins are Widely Represented in Land Plant Genomes

To examine the distribution of TCP proteins in plants, we firstly implemented basic local alignment search tool searches (BLASTP) [27] on 26 Viridiplantae (green plant) genomes in Phytozome 12 [28], BRAD [29], and GENOSCOPE [30] databases, using *A. thaliana* TCP proteins as queries. The 26 Viridiplantae included chlorophytes (*Chlamydomonas reinhardtii*, *Volvox carteri*, *Micromonas pusilla*, and *Ostreococcus lucimarinus*), the liverwort *Marchantia polymorpha*, mosses (*Sphagnum fallax* and *Physcomitrella patens*), the lycophyte *Selaginella moellendorffii*, *Amborella trichopoda*, eudicots (*A. thaliana*, *Brassica napus*, *Brassica rapa*, *Brassica oleracea*, *Citrus sinensis*, *Populus trichocarpa*, *Medicago truncatula*, *Glycine max*, *Eucalyptus grandis*, *Vitis vinifera*, *Solanum lycopersicum*, *Solanum tuberosum*, and *Aquilegia coerulea*), and monocots (*Z. mays*, *Ananas comosus*, *Zostera marina*, and *O. sativa*). We then performed BLASTP searches on another 21 aquatic plant genomes in the National Center for Biotechnology Information (NCBI) database, including Cyanophyta (*Chroococcus* sp., *Microcystis aeruginosa*, *Oscillatoria agardhii*, and *Nostocales cyanobacterium*), Euglenophyta (*Euglena gracilis* and *Colacium sideropus*), Chrysophyta (*Synura petersenii* and *Phaeothamnion confervicola*), Chlorophyta (*Chlamydomonas perpusilla*, *Cosmarium* sp., *Volvox tertius*, *Chlorokybus atmophyticus*, and *Mesostigma viride*), Charophyta (*Chara braunii*, *Chara hispida*, *Chara vulgaris*, *Nitella flexilis*, and *Klebsormidium flaccidum*), Phaeophyta (*Ectocarpus siliculosus* and *Laminaria japonica*), and Rhodophyta (*Porphyra fallax*).

**Figure 1:**
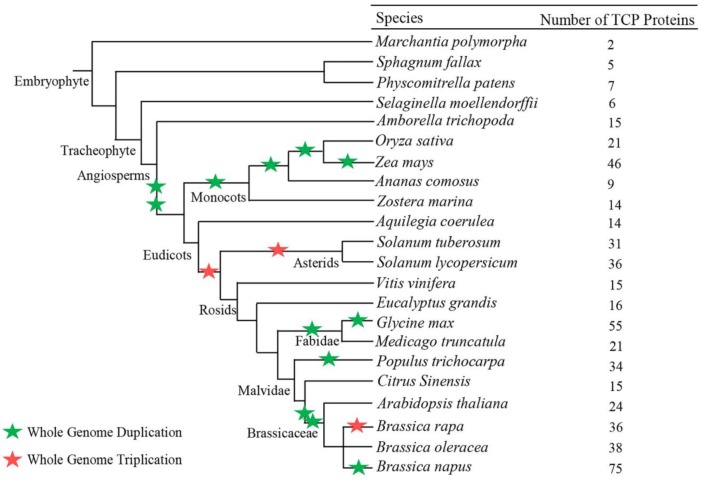
Phylogenetic relationships and total number of TCP (Teosinte-branched 1/Cycloidea/Proliferating) genes for all species investigated in this study with TCP proteins. Phylogenetic relationships among these species have been described in Phytozome (http://www.phytozome.net/) [28]. Green stars indicate whole genome duplication; the red star denotes whole genome triplication (http://chibba.agtec.uga.edu/duplication/).

After eliminating incomplete and redundant sequences, subsequent analyses focused on the proteins with full open reading frames (ORFs). We identified 535 TCP proteins in 22 land plant species. These proteins were present in early-diverging land plants, such as *M. polymorpha*, but none were found in the 25 aquatic plants (Figure 1 and Table S1). One rice TCP protein (LOC_Os07g04510) was excluded from the present study because it had low homology and lacked the TCP domain, but the names of remaining 21 proteins were consistent with that in a previous report [31]. Fifteen and six new genes were identified in maize and tomato, respectively, similar to previous studies [32,33], and thus the corresponding TCP genes in these two species were renamed (Table S1). The genes in the remaining 19 species were first identified in the present study and therefore were named according to their loci on the chromosomes of each species (Table S1). Overall, we identified many candidate TCP proteins in angiosperms and relative few candidate TCP proteins in lower land plants (Figure 1). No TCP protein was identified in aquatic plants (Table S1). In general, there were 14 to 38 TCP proteins in higher plant species, with the allotetraploid *B. napus* showing the largest number of TCP proteins (75), whereas the lower plants *M. polymorpha*, *S. fallax*, *P. patens*, *S. moellendorffii*, and *A. comosus* had only two to nine TCP proteins (Figure 1).

Taken together, our results showed that TCP proteins are present in terrestrial plants only and that these proteins rapidly expanded during the evolution from lower to higher plants, which is consistent with the increasing complexity of land plant genomes.

### 2.2. Classification and Distribution of TCP Proteins in Land Plants

To explore the evolutionary relationships between the 535 TCP proteins in land plants, we constructed a neighbor-joining (NJ) tree based on the alignment of the TCP domains using MEGA 5 [34].

**Figure 2:**
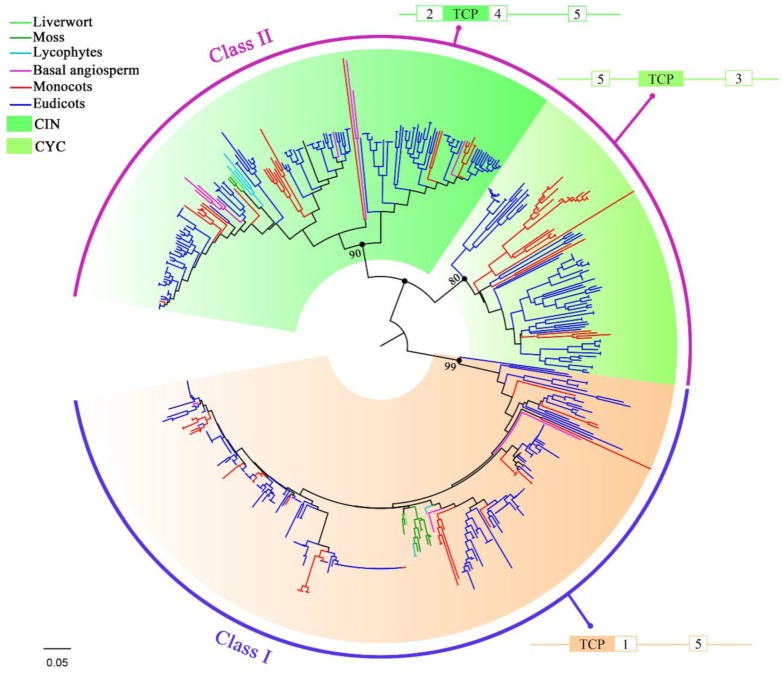
Neighbor joining (NJ) tree of the 535 TCP proteins clustered into Classes I and II. Class II was subdivided into clades CIN and CYC. Colored lines represent different lineages. The peripheral geometric figures indicate the motif structure predicted by MEME [35] in each class or clade.

According to the tree topology and clade support values, the 535 TCP proteins were arranged into Classes I and II with robust bootstrap support (≥60%) (Figure 2 and Figure S1). No species-specific clade was observed, suggesting that the classification and functions of TCP genes were conserved during the evolution of land plants. The Class II was slightly larger than Class I (279 vs. 256 members, respectively) and all of the land plants investigated had TCP gens from both classes, except for *S. fallax*, which had no Class II member. Class II was further divided into the CIN and CYC/TB1 clades based on tree topology and clade support values, with the CIN clade (179 members) being larger than the CYC clade (100 members). All of the land plants investigated had CIN clade TCP genes, while the CYC clade was only found in eudicots and monocots (Figure 1), suggesting that CYC clade is younger than the CIN clade. The CYC genes were absent in the basic angiosperm *A. trichopoda*, indicating that this clade originated from the CIN clade after angiosperm differentiation but before the eudicots and monocots differentiation. However, both Class I and CIN clade genes were present in Embryophyta, suggesting they might have evolved early, although we could not identify which one is older. In our phylogenetic tree, the TCP proteins from each species generally clustered together within a subclade, indicating that they expanded after diverging from their common ancestor. For example, the TCP proteins of mosses (*M. polymorpha*, *S. fallax*, *P. patens*) and *S. moellendorffii* were clustered together into small branches of Classes I and II, respectively, as did the TCP proteins from monocots and eudicots; the TCP proteins of *B. napus* always clustered with those of their ancestral *B. oleracea* and *B. rapa* (Figure S1).

In summary, our phylogenetic analysis revealed that each gene class within the TCP family was conservatively distributed in land plants during their evolution and that Class I and CIN clade of Class II genes were old and emerged at the very beginning of land plants’ establishment.

### 2.3. Conserved Sequence Characteristics within TCP Domains

The DNA-binding domain (DBD) of TFs plays an important role in the recognition and binding of its target gene promoter sequences. To understand the sequence features in the DBD domains of TCP family genes, we performed a comprehensive analysis of the TCP domains across the 535 candidate TCP proteins, based on multiple alignments.

**Figure 3:**
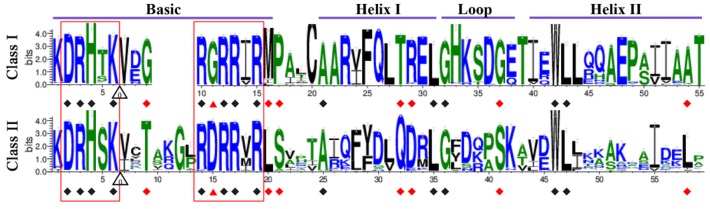
Sequence logos of the plant TCP protein domains. The sequence logos were obtained using the WebLogo online tool (http://weblogo.berkeley.edu/) [40], based on the alignments of the TCP domains. Bit scores indicate information content for each position in the sequence. The black rhomb represents conserved loci (90%) within the whole family. The red rhomb indicates divergent but conserved residues in each subgroup. The red triangle represents the key DNA binding site for the two subgroups. The red box represents the conserved consensus sequence found in all land plants. White triangles indicate the locations of introns, and the number within each triangle indicates the splicing phases of introns.

In general, the TCP domains of candidate proteins in Class I and Class II CIN clade were located at the *N*-terminal. However, the TCP domains of Class II CYC clade proteins were located at the middle of the candidates (Figure 2). There were usually 59 amino acids in the TCP domains of Class II proteins, while in Class I four amino acids were conservatively deleted at the middle of the basic area leading to TCP domains with 55 amino acids (Figure 3). The TCP domains commonly consisted of a basic area and a Helix-Loop-Helix (HLH) structure. The residues in the basic area were highly conserved between Classes I and II, and two consensus sequences, DRHxK and RxRRxR, were found at the *N*- and *C*-terminals of this region, respectively (Figure 3). In *A. thaliana*, it was reported that the glycine (G) residue at the 11th site in the RxRRxR region of Class I and the corresponding residue (aspartate, D) at the 15th site of Class II accounted for the differences in binding targets between these two classes [36]. Moreover, in the same region, the replacement of the arginine residue (R) to threonine (T) at the 15th site of Class I (*AtTCP11* gene) obviously affected protein specificity [18]. Accordingly, we found that the residues at these two sites were highly conserved in Classes I and II (Figure 3), as only a few proteins showed these replacements (1.6% in Class I and 1.8% in Class II), suggesting this region is important for gene functional differentiation. For the HLH region, the residues in Class I TCP proteins were relatively more conserved than in Class II TCP proteins. Only five sites were highly conserved (>90%) in the HLH region between these two classes, including alanine (A)-25, leucine (L)-35, G-36, tryptophan (W)-46, and L-47 in Class II (Figure 3). In contrast, most of the residues within this region were different between these two classes but were highly conserved within the same class (Figure 3), indicating conservative amino acid substitutions in the two classes during the evolution of land plants. Previous studies have also demonstrated that TCP proteins need to dimerize to bind the target DNA, and the second helix (Helix II) of the HLH region is necessary for this dimerization [37]. The LxxLL motif in Helix II has also been reported as involved in protein interaction [11,38]. Accordingly, we found that the residues in Helix II were quite different between Class I and Class II TCP proteins. The first L residue in this motif was generally replaced by isoleucine (I) and valine (V) residues throughout this gene family (Figure 3). The third L residue was partly substituted by an I residue in the CIN clade or by a phenylalanine (F) residue in the CYC clade (Figure S2), indicating that the interactions between Classes I and II may be different. A similar trend was observed within Class II, where members of the CIN and CYC clades generally showed many clade-specific residues, especially in the HLH region (Figure S2), which might be related to their respective functions.

The intron pattern in the DBD of TF gene families is closely related to their evolutionary relationships [39]. Therefore, we further analyzed the intron insertion and evolution patterns in the TCP domains of the 535 candidate TCP genes. In most cases, no intron insertion was observed in the TCP domain; exceptions were the six genes in *Z. marina* (*ZmaTCP7*, *ZmaTCP9*, and *ZmaTCP11* in CIN clade), *S. lycopersicum* (*SlTCP14* in Class I), *S. tuberosum* (*StTCP1* in Class I), and *Z. mays* (*ZmTCP41* in CYC clade). Except for *ZmTCP41*, the other five genes had one conserved intron insertion site and phases (phases 0) in the TCP domains (Figure 3), suggesting that members of the CIN clade were more homologous to that of Class I than CYC members. In addition, we examined the intron insertion events outside the TCP domains, and we found that 115 of the 535 TCP genes (21%) had an intron insertion in this region (Class I: 41 genes; CIN: 31 genes; CYC: 43 genes). The intron insertion sites were mainly located at the *C*-terminals of the TCP genes, and they were conserved in only a few genes of the CYC clade, including *BnTCP1*, *BoTCP32*, *AtTCP18*, *BnTCP37*, *BrTCP2*, *BnTCP72*, *BoTCP21*, *BnTCP12*, *BrTCP16*, and *BnTCP60* with three intron insertions behind the TCP domain, *GmTCP48*, *GmTCP20*, *GmTCP34*, and *GmTCP50* with one intron insertion at the *C*-terminal, and *CsiTCP14*, *GmTCP44*, *GmTCP14*, *GmTCP7*, *GmTCP4*, *MtTCP13*, *PtTCP20*, and *PtTCP25* with one intron insertion at the *C*-terminal.

Together, our results showed that the protein sequence features of the TCP DBD were highly conserved in different classes or clades across land plants, which supported our classification based on the phylogenetic tree. The majority of TCP DBD regions were intron-less and no conserved intron insertion pattern was observed in both of the DBD and non-DBD regions across a class or clade.

### 2.4. Distribution of Non-TCP Motifs Supports the Classification of TCP Genes in Land Plants

In addition to the commonly conserved DBD in a TF gene family, there are usually many conserved non-DBD motifs within a class or clade that are related to gene functions [41]. Therefore, to explore the existence, origin, and evolution of the non-DBD motifs of the TCP gene family across land plants, the MEME tool [35] was applied in this study.

We identified five motifs outside the TCP domain (Figure 2 and Table S2), but only motif 5 was found in Classes I and II of all land plants investigated, indicating it originated from the common ancestor of these two classes. The remaining motifs were distributed in either Class I or Class II, suggesting different evolution pathways for these two classes. Motif 1 was only found in Class I, while motifs 2, 3, and 4 were found in the CIN and/or CYC clades of Class II (Figure 2). Our results also showed that these five motifs were already present in *M. polymorpha*, suggesting they emerged at the same time as the TCP gene family in land plants and then were retained during evolution.

In general, the spatial locations of these motifs were relatively conserved, implying the structure of this gene family was conserved during land plants’ evolution. However, motif 5 frequently changed its location to either before (in CYC clade) or after (in CIN clade and Class I) the TCP domains (Figure 2). Moreover, motifs 2 and 4 in the CIN clade of Class II and motif 1 in Class I were adjacent to the TCP domains, with motif 2 adjacent to the *N*-terminal of the TCP domain and motifs 1 and 4 adjacent to the *C*-terminal instead, indicating these motifs co-evolved with the TCP domain (Figure 2). However, no motif was adjacent to the TCP domains in the CYC clade of Class II. Thus, the structure of TCP proteins in the CIN clade of Class II is more similar to that of Class I TCP proteins than to that of TCP protein in the CYC clade of Class II, implying that the CIN clade is older than the CYC clade.

Overall, our results demonstrated that the protein structure of closely related members in a given clade were remarkably conserved across land plants, and that CIN and Class I TCP proteins were more similar in both motif distribution and structure, implying a common origin or close relationship.

### 2.5. Rapid Expansion of TCP Genes in Land Plants

As mentioned above, the large number of TCP genes found in higher plants indicates this gene family underwent rapid expansion in land plant genomes during their evolution. To further examine the expansion mechanism of TCP genes, we used CoGe online software [42] to determine the syntenic relationship among the TCP gene candidates in 22 land plants.

**Figure 4:**
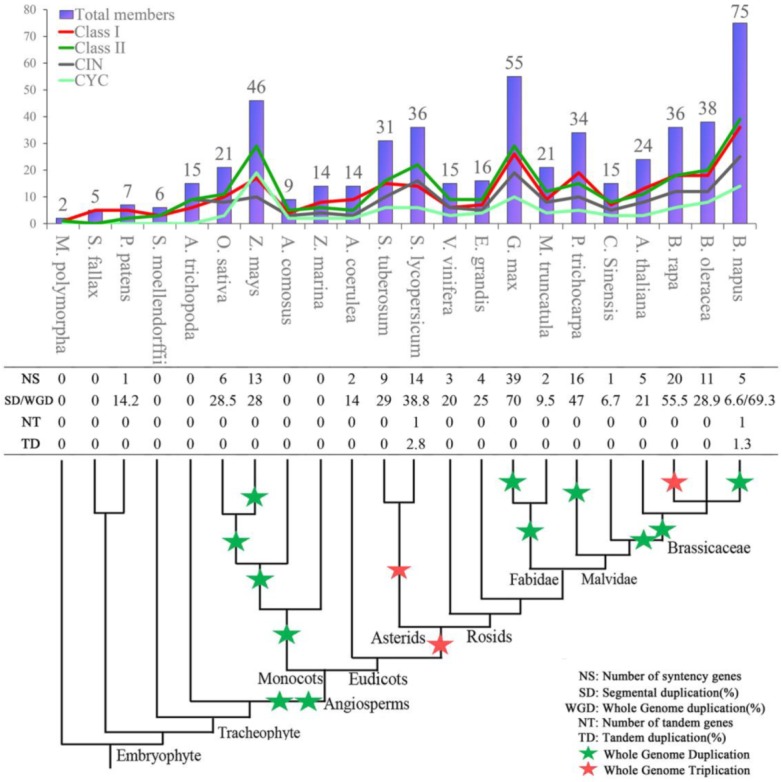
Number of duplications in land plant TCP genes. The histogram displays the number of TCP proteins in each species. Phylogenetic relationships among these species have been described in Phytozome [31]. Green and red stars indicate whole genome duplication and triplication in the corresponding species, respectively.

Many TCP genes had close syntenic relationships, and the segmental duplication rate in most land plants varied from 5% to 30% (Figure 4). Moreover, we found that species that experienced whole genome duplication or triplication (Figure 4) seemed to have more syntenic duplication genes than species that did not experience such events; in *G. max*, *B. rapa*, and *B. napus* syntenic duplication genes were ˃50%. Furthermore, most segmental duplication genes may be formed in ancestral genomes and be retained during whole-genome replication events. For example, in *B. napus* (AACC; *n* = 19), most TCP genes (45.3%) derived from the recent hybridization event of the two ancestor diploid genomes (*Brassica rapa* (AA; *n* = 10) and *Brassica oleracea* (CC; *n* = 9)) ~75 million years ago, while only 6.6% genes originated from segmental duplication. Similar situations were observed in *B. rapa* and *G. max*. Polyploidy is a remarkable characteristic of higher plants’ chromosome evolution, and most extant angiosperms have experienced polyploidization at least once during their evolution [43]. Accordingly, our results showed that the rapid expansion of the TCP gene family was consistent with the polyploidization trend of land plants (Figure 4). Thus, we hypothesized that the segmentally duplicated genes, either inherited from ancestor hybridizations (whole genome duplication) or produced within a plant genome, were the major evolutionary force for TCP gene family expansion. Contrarily, tandem duplication events were identified in only three species, *S. lycopersicum*, *G. max*, and *B. napus* (Figure 4), demonstrating it contributed little to the expansion of this gene family.

Overall, whole-genome replication and segmental duplication seem to be the major drivers for TCP gene family expansion.

### 2.6. MicroRNA319 Targets of TCP Genes were Conserved in Angiosperms

MicroRNAs bind to specific genes as negative regulators, thus playing an important role in the regulation of the expressions of TF genes in plants [44]. To explore the possible relationship between microRNAs and TCP genes, we performed a comprehensive prediction of microRNA-target genes across the 22 land plants investigated in this study on the psRNATarget website (http://plantgrn.noble.org/psRNATarget/).

Sixty microRNAs were predicted to have a relationship with TCP genes (Table S3). In general, most microRNAs targeted only a few genes in each class. However, microRNA319 (miR319) targeted 90 TCP genes across 18 angiosperm species (Table S3), which was consistent with previous studies [41,45]. In plants, miR319 is one of the most conserved and ancient microRNA families, and it is found in diverse plant species from moss to flowering plants [46,47]. Although TCP genes were found in lower land plants, such as *M. polymorpha*, *P. patens*, and *S. moellendorffii*, the miR319 target genes were observed at the earliest in the basal angiosperm *A. trichopoda* (Table S3), indicating that this regulatory relationship evolved relatively late in land plants. Moreover, the majority of the miR319-target genes were clustered in a subclade of the CIN clade (homologs of *AtTCP2*, *AtTCP3*, *AtTCP4*, *AtTCP10*, and *AtTCP24*), with only one *G. max* gene (*GmTCP5*) outside this subclade (Figure S1), indicating that this regulatory relationship is conserved in angiosperms. Interestingly, many *S. lycopersicum* and *S. tuberosum* TCP genes in the same subclade lost the miR319 target site, suggesting a different regulatory mechanism in this lineage.

It was reported that, during evolution, microRNA-target genes with relaxed selective pressure usually have more members than non-microRNA-target genes [48]. However, our results showed that the synonymous/non-synonymous mutations (Ka/Ks) ratio of the miR319-target and non-miR319-target TCP genes in the CIN clade were lower than 1.0 (Table S4), and there was no obvious difference between them, although the miR319-target genes showed relatively lower selective pressure.

Overall, our results suggested that the relationship between miR319-target genes and TCP genes was conserved in angiosperms.

### 2.7. The Expressions of TCP Genes in Land Plants Suggest Function Diversification and Conservation

To examine the temporal and spatial expressions of TCP genes across land plants, we compared their expression patterns in representative land plant lineages at the different developmental stages, including *M. polymorpha*, *P. patens*, *B. napus*, *G. max*, *Z. mays*, and *O. sativa*.

In lower plants, the *M. polymorpha* gene *MpTCP2* (Class I) had high expression levels in the archegoniophore, sporophyte, sporeling, and thallus, whereas *MpTCP1* (Class II) had high expression levels in the antheridiophore, sporophyte, thallus, and gametophore (Figure S3A). In *P. patens*, TCP genes within both classes were mainly expressed in the reproductive tissues and Class I genes were also expressed in the protoplast (Figure S3B). These results suggested that the TCP genes might be primarily involved in the regulation of leaf and reproductive development.

**Figure 5:**
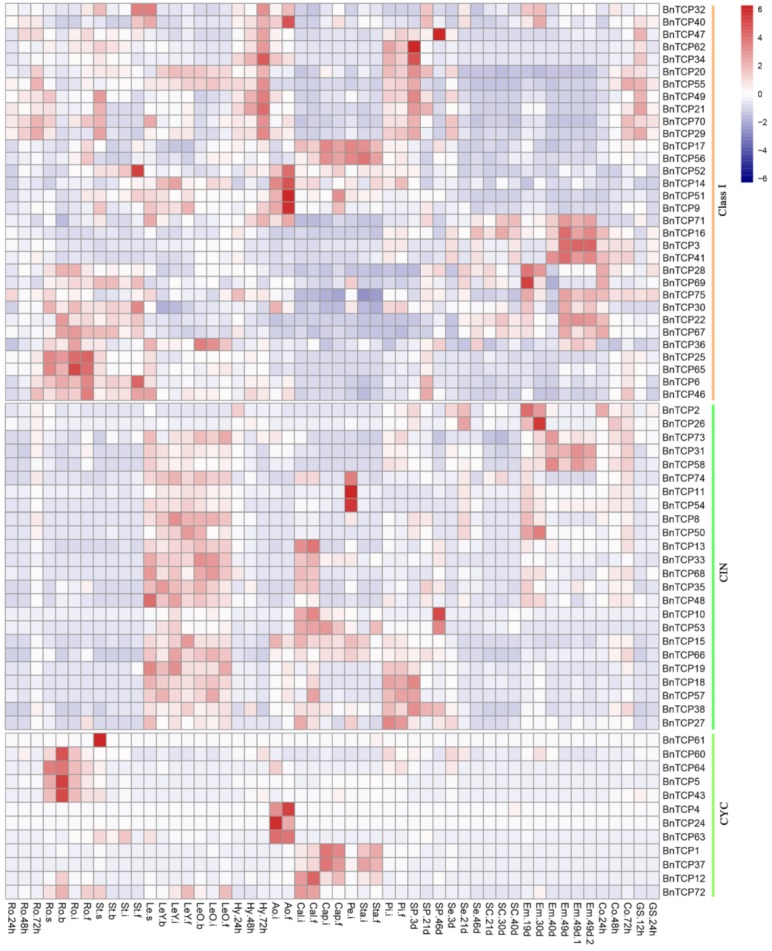
Expression profiles of TCP genes in *Brassica napus* across different developmental stages and organs. The heatmap was drawn using the R package [52]. Genes and their subgroups are shown on the right. Tissues used for expression analysis are indicated at the top of each column. Ro: root; St: stem; Le: leaf; Hy: hypocotyl; Ao: anthocaulus; Cal: calyx; Cap: capillament; Pe: petal; Sta: stamen; Pi: pistil; Sp: silique pericarp; Se: seed; Sc: seed coat; Em: embryo; Co: cotyledon; and GS: germinating seeds. D; days; H: hours. “-s”, “-b”, “-i”, and “-f” represent the “seedling”, “bud”, “initial flowering”, and “full bloom” stages of *B. napus,* respectively. Colored bars represent log2 expression values. Red, green, and black indicate high, low, and medium gene expression levels, respectively.

In eudicots, most of the *B. napus* TCP genes were expressed in the various stages of vegetative and reproductive organ development at different levels (Figure 5). All Class I genes except *BnTCP7*, *BnTCP44*, and *BnTCP45* had high expression levels in many tissues or organs across various development stages. The same trend was observed for *G. max* Class I genes (Figure S3C). These findings corroborated the functions of Class I TCP genes in plant differentiation, leaf and flower development, and seed germination [6,20,49-51]. For Class II, almost all of the CIN genes in *B. napus* (Figure 5) were highly expressed in the leaf. This was consistent with their well-known functions in regulating leaf development [45]. Some Class II genes also had high expression levels in flower and seed tissues. The *G. max* CIN genes had relatively wide expression profiles and they were highly expressed in leaf, flower, and seed tissues (Figure S3C). In contrast, the CYC genes were expressed in a few tissues or organs, being highly expressed in *B. napus* root and flower and in *G. max* leaf, flower, and seed (Figure 5 and Figure S3C). Similar expression profiles of Class I and Class II TCP genes were observed in *B. rapa*, *Citrullus lanatus*, and tomato [30,49,50], suggesting that their roles were conserved in dicots.

In monocots, like *O. sativa* (Figure S3D), the Class I and Class II CIN genes were extensively expressed in all tissues investigated. On the other hand, the CYC genes were only expressed in panicle, seedling, and endosperm tissues. In *Z. mays* (Figure S3E), the Class I and Class II CIN genes were also highly expressed in many tissues and/or organs. Eleven of the 19 maize CYC genes were not expressed in any tissue, and the remaining eight were only expressed in tassel, seed husk, and embryo tissues. The similarities in the expression patterns of TCP genes in *O. sativa* and *Z. mays* suggested the functional conservation of TCP genes in monocots.

Taken together, our results showed that the expression profiles of TCP genes were generally broadened during land plant evolution. Extended expression profiles usually occurred in newly or more recently evolved forms. For example, Class I genes were mainly expressed in the leaf and reproductive tissues of moss, but they were highly expressed in the leaf, flower, and seed tissues of dicots. This is consistent with previous studies in which Class I genes were suggested to play important roles in plant evolution [16,51]. The Class I and Class II CIN clade genes showed similar expression trends in many tissues across all land plants, suggesting the ancestral role of TCP genes in the regulation of leaf and reproductive tissue development. The CYC Class II clade genes had limited expression, and thus they might perform minor and specific roles in plants.

### 2.8. Diversified and Conserved Protein Interaction Network of TCP Proteins in Land Plants

In plants, TCP proteins were reported to function in many biological processes, such as plant immunity [37,53], leaf development [19], and floral transition [54], by forming protein complexes. Thus, the investigation of the interaction network of TCP proteins among land plants can help us understand the function and evolution mechanism of TCP proteins during evolution. Here, we used STRING database [55] to compare the interaction networks of TCP proteins in *P. patens*, *A. thaliana*, *G. max*, *O. sativa,* and *Z. mays.*

Our results showed that there were significantly more protein interaction relationships in Class I than in Class II TCP proteins. For example, for *A. thaliana*, there were 348 protein interaction pairs in Class I but only 201 pairs in Class II (CIN and CYC clades) (Tables S5 and S6). The major interaction proteins of Class I were involved in phytohormone signaling, plant immunity, circadian clock, photoperiod, transcriptional initiation, and ribosome formation processes, and this was consistent with the diverse functions and extensive expression profiles of TCP genes. Among these numerous interacting proteins, we found that the interaction TCP proteins of Class I involved in the gibberellin (GA) signal pathway were conserved from mosses to higher plants, suggesting this interaction relationship originated early and it was conserved during land plants’ evolution (Figure S4, Tables S5 and S6).

Many protein interactions were also found for the CIN clade of Class II (Tables S5 and S6), and these were mainly involved in leaf development, circadian clock, phytohormone signaling, transcriptional initiation, and protein folding. The CIN genes are known to regulate leaf development. In *A. thaliana*, it was reported that *AtTCP2*, *AtTCP3*, *AtTCP4*, *AtTCP10*, and *AtTCP24* genes may indirectly regulate leaf development by regulating the boundary-specific genes *CUC* and *LOB* [56]. We found that these CIN proteins interact with AS2 and ABAP1, which participate in leaf development (Figure S5A, Tables S5 and S6), indicating the possible roles of these TCP genes in this process. The functions of most predicted protein interactions in *G. max*, *O. sativa*, *Z. mays*, and *P. paten* remain unknown (Figure S5B–D, Tables S5 and S6). Nevertheless, the homeobox domain-containing proteins OSH1 in *O. sativa* and RS1 in *Z. mays* involved in leaf development were found to interact with CIN proteins. Thus, CIN proteins may have similar functions in the regulation of leaf development in different species.

**Figure 6:**
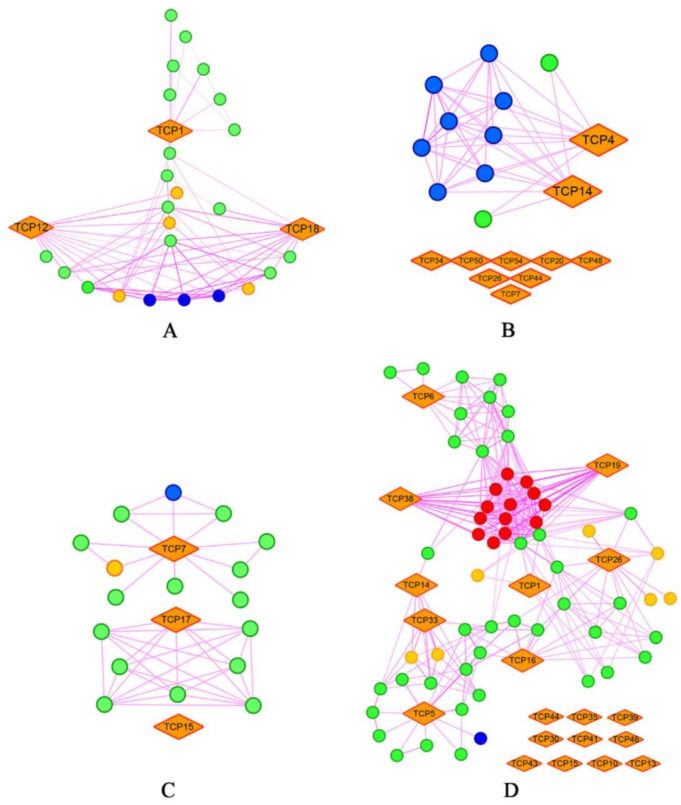
Interaction network of TCP proteins within the CYC clade in (**A**) *Arabidopsis thaliana*, (**B**) *Glycine max*, (**C**) *Oryza sativa*, and (**D**) *Zea mays* according to the STRING dataset [55]. The interaction network was constructed in Cytoscape 3.6.1 [62]. CYC proteins are displayed as orange rhombs. Light orange circles represent other TCP proteins. Blue circles indicate proteins involved in the strigolactone pathway. Red circles represent the Hsp70 domain-containing proteins.

In the CYC clade, interaction TCP proteins were mainly involved in phytohormone biosynthesis and signaling, molecular chaperones, cell cycle, and flower development (Tables S5 and S6). The CYC proteins are considered important regulators of plant architecture. According to previous studies, the roles of CYC proteins in the regulation of plant branching patterns were conserved in different species [2,17,57,58]. In the present study, we found that the interaction model for branching was different between monocots and dicots. For instance, in both *A. thaliana* and *G. max*, CYC proteins interacted with many proteins involved in the strigolactone (SL) pathway (Figure 6A,B) while in *Z. mays* and *O. sativa*, only a few proteins in the SL pathway were found to form protein complexes with CYC proteins (Figure 6C,D). This has been confirmed in *A. thaliana* and *Z. mays*, as AtBRC1 was reported to be a key regulator of SL downstream [2] while ZmTB1 was independent from SL signaling [59] and its interaction with downstream genes differed from those of AtBRC1 [60,61]. Therefore, CYC proteins may participate in the regulation of branching via distinct pathways in different species. Moreover, the *O. sativa* and *Z. mays* CYC proteins did not cluster perfectly with the dicots CYC proteins in our phylogenetic analysis, corroborating the functional divergence between this type of proteins in dicots and monocots. Furthermore, *Z. mays* CYC proteins, ZmTCP19, and ZmTCP38 were found to interact with many HSP70 proteins (Figure 6D). These results suggested that CYC genes functionally differentiated in the distinct species during land plants’ evolution. Further research on CYC proteins will help us to better understand the evolutionary history of plant architecture and genetically control plant architecture.

Overall, we identified numerous interactions for TCP proteins. Class I and Class II CIN proteins have more interaction proteins than CYC proteins, and Class I proteins tend to interact with CIN rather than CYC proteins. Further studies of these protein interactions will help us elucidate the roles of TCP proteins in plants.

## 3. Discussion

### 3.1. Similar, Antagonistic, and Evolutionary Functions of TCP Proteins in Plants

The TCP family is known to be involved in plant development, and recently, it was discovered to be involved in hormonal pathways and plant defense. Here, we summarized the functions of plant TCP proteins (Table 1).

To date, the functional studies on this gene family mainly focused on Class II members, and they were reported to take part in plant development. For Class II members, those in the CIN clade are mainly involved in leaf development. For instance, in *A. thaliana*, *AtTCP2*, *AtTCP3*, *AtTCP4*, and *AtTCP10* genes are involved in leaf edge development [41]; *AtTCP3* indirectly regulates the expression of the boundary-specific genes *CUC* and *LOB* [56]; and a CIN homologous gene, *LA*, in *S. lycopersicum* regulates compound-leaf formation [45]. Moreover, a few CIN homologous genes were reported to function in cell proliferation [63], photoperiodic flowering [64], floral organ development [65], and cold stress and defense [66,67]. Members of the CYC clade regulate branching, as is the case of *AtBRC1* in *A. thaliana* [2], *OsTB1* in rice [1], and *PsBRC1* in *Pisum sativum* [57]; some genes are important for flower development [68,69,70] as well. Together, these results support that the genes in CIN and CYC clades are mainly involved in plant development, although those in the CIN clade have wider functions, consistent with their expression profiles in leaf and flower tissues obtained in this study (Figure 5). These results implied that the roles of genes within these two clades may be different.

Studies on Class I members are relatively fewer than those on Class II members. Class I members are also involved in regulating plant development, but have an opposite effect to those of Class II. Their best-known function is the regulation of leaf development: whereas Class I genes promote cell proliferation, CIN genes of Class II negatively regulate this process [71,72]. Moreover, Class I members function in many other development processes. For example, *A. thaliana AtTCP14* and *AtTCP15* genes play roles in cell proliferation in internode, leaf, and floral tissues, and affect internode length and leaf shape [3]; the *Chrysanthemum morifolium CmTCP14* gene regulates organ size [73], the *Phalaenopsis equestris PePCF10* gene affects ovule development [74], and the *Gossypium hirsutum GhTCP14* gene is involved in auxin-mediated cotton fiber development [75]. Recently, Class I members were found to participate in root nitrate absorption [76], and demonstrated to participate in biotic and abiotic stresses. For example, in *O. sativa*, *OsTCP19* enhances responses to abscisic acid (ABA) response, and salt, drought, and cold stresses [77] while *OsPCF2* and *OsPCF6* participate in the regulation of salt stresses [76,78]. In *A. thaliana*, *AtTCP14* and *AtTCP15* were involved in defense responses against *Hpa* [53] and DC3000 pathogens [37]. The diverse functions of this class in many developmental processes are consistent with their wide expression profiles in our analysis.

It is interesting that both Class I and Class II genes were involved in hormone pathways, but they usually acted in opposite directions. For example, in *A. thaliana*, *AtTCP20* in Class I promotes auxin biosynthesis by promoting the expression of *NRT.1* [79], while *AtTCP3* in CIN clade of Class II negatively modulates auxin response [80]; *AtTCP20* also inhibits the jasmonic acid (JA) biosynthesis [19], whereas *AtTCP4* in CIN clade affects JA biosynthesis in the opposite direction [81]. Antagonism between Class I and Class II genes was also observed in cytokinin (CK), ABA, and GA [5] pathways.

Recent studies revealed that TCP genes play crucial roles in plant immunity. For example, in *A. thaliana,* some TCP genes, such as *AtTCP13* in the CIN clade and *AtTCP14*, *15*, *19*, and *21* in Class I, act as pathogen effector targets [82]. In *O. sativa*, *OsTCP21* in the CIN clade participates in pathogen defense [67]. In *S. lycopersicum*, *TCP14-2* (ortholog of *AtTCP14*) also contributed to enhance immunity to *Phytophthora capsici* [83]. Meanwhile, many TCP genes in Class II have been shown to be targets of phytoplasma effectors, SAP11 and its homologs [84,85,86]. For instance, in *A. thaliana,* SAP11 interacts with and destabilizes different members of CIN clade in class II, leading to the induction of severe leaf crinkling and down-regulation of jasmonic acid (JA) synthesis [84,87]. Recently, members of CYC clade in Class II were also demonstrated to be the targets of SAP11-like effectors (e.g., SWP1 from wheat blue dwarf phytoplasma) [88,89]. Notably, there are other kinds of phytoplasma effectors, such as TENGU, only SAP11 and its homologs target TCP proteins [84,89]. To date, the domains and/or sites of TCP proteins that are required for the interaction with the pathogen effectors remain unknown. However, given the fact that the protein architectures and sequence characteristics of TCP proteins are remarkably conserved in each class or clade (Figure 2 and Figure 3) and that SAP11 effectors are present in a range of phylogenetically distant phytoplasmas [88], it is likely that SAP11-mediated TCP factor degradation may exist in a wide-range lineages and is conserved during evolution.

In summary, TCP genes are mainly involved in plant development, hormonal processes, and plant defense. The Class I and the CIN clade Class II genes had numerous functions whereas the CYC clade Class II genes had fewer roles. Moreover, the functions and expressions of CIN and CYC genes were obviously different, suggesting their functional divergence. Nevertheless, the Class I genes usually had opposite effect to Class II genes and this antagonism may be common in the plant kingdom during the process of plant evolution.

### 3.2. Origin and Evolution of the TCP Gene Family in Land Plants

**Figure 7:**
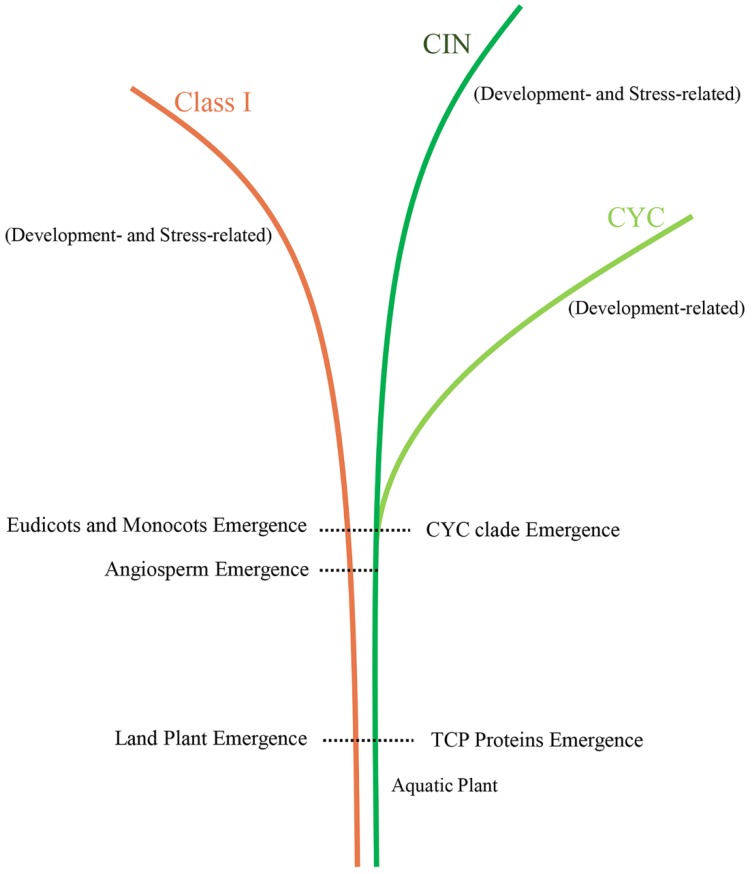
Evolutionary scenario of TCP genes in land plants. Dotted lines indicate the absence of TCP proteins in aquatic plants.

In this study, we assessed the origin and evolutionary relationships of the TCP gene family across 47 plants ranging from algae to angiosperms using homology search of full-length proteins. Therefore, we could propose an evolutionary scenario for the TCP gene family (Figure 7).

Previously, it was reported that this gene family may have originated in algae by the homologous cloning method [12]. However, we did not identify any sequence corresponding to TCP genes in the same species [12]. Similarly, no TCP gene was identified in any of the 25 algae genomes investigated in the present study, including the freshwater algae *Klebsormidium nitens* genome (Table S1). However, the TCP genes were found in the genome of basal land plants such as *M. polymorpha* (Figure 1). Therefore, TCP genes may have originated early in the history of land plants, after plants migrated from water to land more than 450 million years ago [109]. Given their roles in plant development, the significant lifestyle changes of land plants may have contributed to the appearance of TCP genes, as these promote the development of new organs (e.g., leaf, branching, and flower) enhancing plants’ ability to adapt to the (new) terrestrial environment [110]. Embryogenesis is characteristic of land plants, and our expression profile analysis suggested that TCP genes may regulate early thallus development and embryogenesis (Figure S4), also supporting the origin of TCP genes in the early history of land plants.

Most of the previous investigation in individual plant species [31,33,111,112] generally divided TCP family genes into Classes I and II, with Class II being further divided into CIN and CYC clades, but some studies [26,113,114] divided this gene family into nine to 11 subgroups. In our analyses, consistent with most of the previous studies, the topology of the phylogenetic tree of the 535 plant TCP proteins was preliminary divided into two major classes, namely Class I and II (Figure 2). However, the support value of Class I was low (<50%), and Class II was obviously divided into two clades (CIN and CYC clades) (Figure S1). Given the known functions (Table S7), expression profiles (Figure 5), protein structure (Figure 2), and sequence features in the DBD regions (Figure 3), the TCP genes within CIN and CYC clades were obviously different from each other but were conserved in each clade, suggesting that they had gone through functional differentiation during evolution. Therefore, we suggest to classify the TCP gene family into three major classes, Class I, Class II (CIN), and Class III (CYC). As members of Class I and Class II (CIN) were found in *M. polymorpha*, while those of Class III (CYC) were found in monocots and dicots, the CYC clade might have derived from the CIN clade before the divergence of dicots and monocots. Moreover, a recent study reported that *PpTCP5* (CIN gene) is involved in regulating sporangium branching in *P. patens* [99], similar to the well-known function of members of the CYC clade in plant branching. Thus, the regulatory roles of CYC genes in branching might have evolved early, supporting our hypothesis that the CYC clade originated from the CIN clade. A previous study also showed that three major duplication events before the core dicot differentiation produced three types of CYC genes [13]. A later study confirmed these duplications events in basal dicots [115]. Our phylogenetic analysis also confirmed these three major gene duplications in the CYC clade in core dicots (Figure S1), and we found that the TCP genes in Classes I and II underwent many duplications in both dicots and monocots during land plants’ evolution (Figure 4 and Figure S1). Furthermore, in the CYC clade, the monocot homologs were obviously separated from the dicot homologs (Figure S1), which suggests that their duplications mainly occurred after the dicot and monocot differentiation. However, these duplications in the CIN clade and Class I TCP genes were different, as the homologs of dicots and monocots were generally clustered together (Figure S1). These results suggested that these genes were relatively more conserved than those within the CYC clade during evolution, which might be due to the functional differentiation of CYC clade genes during evolution. Based on our results, we hypothesize that two important stages characterize the evolution of the TCP gene family. The first stage occurred early in land plant history when the TCP genes first appeared. The second occurred before the differentiation of monocots and dicots when the CYC clade derived from the CIN clade and it was followed by different expansion and/or evolution trends in the CYC clade between dicots and monocots.

As plant complexity increased during the evolution, more intricate regulatory networks were needed. Gene duplication leads to the increase in the number of genes, thus providing the raw genetic resources for natural selection. Previous studies showed that segmental duplication contributed to the expansion of the TCP gene family [114]. However, our results revealed that the increasing trend of TCP genes in plant genomes is highly associated with that of increasing complexity and whole-genome duplication events of plant species (Figure 4), evidencing that whole-genome duplication was the major driver of TCP gene expansion across land plants instead of segmental duplication. After duplication, most TCP genes were retained in new species. For example, among the 75 TCP genes in *B. napus*, 52 genes (69%) derived from *B. rapa* (33 genes) and *B. oleracea* (19 genes) by a recent hybridization (about 75 million years ago) between these two ancestors. This is consistent with previous studies in which TF genes were preferentially retained after duplications during evolution [39,116]. Moreover, we found that most TCP genes from *B. rapa* (33 genes, 92%) were maintained in *B. napus* while 50% genes from *B. oleracea* were lost, suggesting the tendency of gene retention after whole-genome duplication. Taken together, our results revealed that TCP genes mainly expanded through whole-genome duplication, and most of the duplicates were retained during land plants’ evolution.

## 4. Materials and Methods

### 4.1. Sequence Retrieval

To explore the evolutionary relationships of the TCP gene family among land plants, BLASTP [27] searches were conducted for 26 sequenced plant genomes ranging from unicellular green algae to multicellular flowering plants registered in Phytozome (http://www.Phytozome.net) [28]. For each lineage, ≥1–2 species with relatively well assembled and annotated genomes were selected as representatives. To ensure that no TCP proteins were inadvertently eliminated by lack of correspondence to the consensus, representative sequences of *A. thaliana* TCP proteins [24] were used as queries (Table S1), and a low-stringency criterion was applied (cutoff *p* < 0.1). After deleting the partial and redundant sequences, the TCP domains were identified in the candidates using ExPASy (http://expasy.org/prosite/) [117]. The same procedure and criteria were applied to identify TCP proteins in *B. oleracea* (v 1.1) in BRAD [29] and *B. napus* (v 1.0) in GENOSCOPE (http://www.genoscope.cns.fr/brassicanapus/) [30]. Moreover, to confirm the origin of the TCP genes in lower plant species, a BLASTP search was performed on another 21 aquatic algae in NCBI (https://blast.ncbi.nlm.nih.gov/Blast.cgi) (Table S1). For balanced and unbiased dataset representation, candidates were considered positive if their proteins contained at least a relatively complete TCP domain. All newly identified sequences were named according to protein identifiers from each genome (Table S1).

### 4.2. Multiple Sequence Alignments and Gene Structure Analysis

Multiple alignments of the TCP domains of candidate proteins were performed in MAFFT 5 using the default parameters [34]. Intron insertion information for the candidate genes was acquired from the corresponding genome databases. Conserved non-TCP-domain protein motifs were identified with MEME [35]. The following settings were used: maximum number of motifs: 20; minimum motif width: 6; and maximum motif width: 100. All putative motifs with expected values >1 × 10^−100^ 1E-100 and shared by about 70% members in a given class or clade were retained.

### 4.3. Phylogenetic Analysis

A NJ tree was constructed in MEGA 5 [34] based on the alignment of the TCP domains. To determine statistical reliability, a bootstrap analysis was applied with 1000 replicates, using p-distance and pairwise deletion.

### 4.4. Genome Synteny and Gene Duplication Analyses

Syntenic blocks among the land plant species investigated in this study were downloaded from the PGDD (http://chibba.agtec.uga.edu/duplication/) [118], BRAD (http://brassicadb.org/brad/) [29], and CoGe (https://genomevolution.org/coge/) [42] databases. All candidate TCP genes were mapped to the syntenic blocks for intra- and inter-genomic comparisons. Tandem duplication genes were identified according to their physical locations within individual chromosomes and ≤1 intervening gene.

### 4.5. Gene Expression Analysis

The expression datasets of *Z. mays* (accession number: GSE27004) and *O. sativa* (accession number: GSE19024) were obtained from the Gene Expression Omnibus (GEO, https://www.ncbi.nlm.nih.gov/geo/). The *B. napus* expression datasets were downloaded from the BioProject PRJNA358784 (NCBI database). The *G. max* expression dataset was obtained from Soybase (https://soybase.org/soyseq/). The *P. patens* expression dataset was acquired from BAR (http://bar.utoronto.ca/). The *M. polymorpha* expression dataset used was retrieved from a previous study [119]. The heatmap was drawn using the R package [52]. All genes with fragments per kilobase of exon model per million reads mapped (FPKM) <1 were excluded from the heatmap.

### 4.6. Interaction Network and miR319-Target Analysis

The interaction network of TCP proteins was constructed based on the STRING 10.5 database (http://www.string-db.org) [55] and displayed using Cytoscape 3.6.1 [62]. To predict microRNA target genes, the full lengths of candidate TCP coding sequences were analyzed using the psRNATarget website [120]. The predicted target relationships with expectation >3 were excluded from our results. The nucleotide substitution rate (Ka/Ks) of miR319-target and non-miR319-target genes was calculated using the Ka/Ks online tool (http://services.cbu.uib.no/tools/kaks).

## Figures and Tables

**Table 1:** Summary of the functionally characterized plant TCP genes found in the present study.

Class (Clade)	Species	Name	Biological Function
Class I	*Brassica rapa*	*BrpTCP4*	Head shape from round to cylindrical [90]. Plant immunity [84].
	*Arabidopsis thaliana*	*AtTCP15*	Regulate the expression of the key cell cycle genes [20]; regulate the cytokinin and auxin response [4].
		*AtTCP14, 15*	Influence the internode length and leaf shape [3]. Related to the defense of *Hpa* pathogen [53] and DC3000 [37]. Plant immunity [82].
		*AtTCP14*	Involved in seed germination [6].
		*AtTCP16*	Regulate meristem induction and differentiation [91]. Involved in Pollen development [92].
		*AtTCP20*	Involved in cell proliferation, division and differentiation [93]. Regulate leaf pavement cell sizes and senescence [19]. Mediate nitrate foraging by Arabidopsis roots [94]. Influence the absorption of nitrate [74].
		*AtTCP19, 20*	Control leaf senescence [95].
		*AtTCP21*	Regulate circadian clock activity [7]. Plant immunity [82].
		*AtTCP23*	Related to flowering time and plant development [21].
		*AtTCP8*	Regulate SA biosynthesis and signal transduction [96].
	*Gossypium hirsutum*	*GhTCP14*	Regulate cotton fiber cells development [73].
	*Oryza sativa*	*OsTCP19*	Involved in salinity and drought tolerance [75].
		*PCF2*	Involved in salt stress tolerance [78].
	*Phalaenopsis equestris*	*PePCF10*	Involved in leaf and ovule development [72].
	*Chrysanthemum morifolium*	*CmTCP14*	Inhibited organ size and delayed senescence [71].
Class II (CIN)	*Arabidopsis thaliana*	*AtTCP17*	Up-regulate phytochrome interaction factors and auxin biosynthesis genes to avoid shade [97].
		*AtTCP4*	Involved in trichome differentiation and photoperiodic flowering [63,64]. Promote JA biosynthesis involving in leaf development and senescence [81].
		*AtTCP24*	Regulate anther wall development [65].
		*AtTCP3*	Promote flavonoid biosynthesis and negatively regulate auxin response [80]. Regulate flower morphology [56].
		*AtTCP13*	Involved in light response [98].
		*AtTCP2, 3, 4, 10*	Involved in leaf development [41]. Plant immunity [84].
	*Oryza sativa*	*OsTCP21, PCF6*	Involved in cold stress tolerance and plant defense [66,67].
	*Solanum lycopersicum*	*LA*	Involved in leaf development [45].
	*Phalaenopsis equestris*	*PeCIN8*	Regulate ovule, leaf, and petal development [72].
	*Physcomitrella patens*	*PpTCP5*	Negatively regulate sporophyte branching [99].
Class II (CYC)	*Arabidopsis thaliana*	*AtTCP18*	Suppresses the growth of axillary bud [2]. Repress the floral transition [54]. Plant immunity [89].
		*AtTCP1*	Influence petioles, rosette leaves, and inflorescent stems development [100].
	*Cucumis melon*	*CmTCP1*	Involved in development of tendrils from lateral shoots [101].
	*Cucumis sativus*	*TEN*	Regulate tendril-less phenotype [102].
	*Gerbera hybrida*	*GhCYC2*	Partake in flower symmetry [66].
	*Pisum sativum*	*PsBRC1*	Regulate shoot branching [57].
	*Solanum lycopersicum*	*SlBRC1b*	Suppress shoot branching [58].
	*Solanum tuberosum*	*BRC1a*	Regulate lateral branching [103].
	*Hordeum vulgare*	*INT-C*	Regulate tillering and fertility of lateral spikelets [104].
	*Oryza sativa*	*FC1*	Influence plant height tillering [1].
		*REP1*	Control palea development and floral zygomorphy [67].
	*Sorghum bicolor*	*SbTB1*	Negatively regulate tillering [105].
	*Switchgrass*	*PvTB1*	Negatively regulate tillering [106].
	*Zea mays*	*BAD1*	Regulate inflorescence architecture [107].
		*TB1*	Negatively regulates tillering [9].
	*Tulipa gesneriana*	*TgTB1*	Suppress the growth of axillary bud [17].
	*Chrysanthemum morifolium*	*CmCYC2c*	Regulate petal development [108].
	*Lotus japonicus*	*LjCYC5*	Regulate the symmetry of the inflorescence [68].

## Supplementary Materials

Supplementary materials can be found at https://www.mdpi.com/1422-0067/20/14/3591/s1. Table S1: Candidate TCP genes identified in land plants in the present study. Table S2: Protein motifs of TCP proteins in the land plants examined in the present study. Table S3: Predicted microRNA-target site of TCP genes in the land plants examined in the present study. Table S4: Nucleotide substitution rate (Ka/Ks) of miR319-target and non-miR319-target genes. Table S5: Details of the TCP protein interaction network in *Arabidopsis thaliana*. *Glycine max*, *Oryza sativa*, *Zea mays*, and *Physcomitrella patens*. Table S6: The annotation of proteins in the interaction network of TCP proteins in *A. thaliana*, *G. max*, *O. sativa*, *Z. mays*, and *P. patens*. Figure S1: Phylogenetic tree of the 535 TCP proteins used in the present study. Figure S2: Sequence logos of the TCP domains in Class II TCP proteins. Figure S3: Expression profiles of TCP genes in *Marchantia. polymorpha*, *P. patens*, *G. max*, *Z. mays*, and *O. sativa* across different developmental stages and organs. Figure S4: Protein interaction networks of Class I TCP proteins in (A) *A. thaliana*, (B) *G. max*, (C) *O. sativa*, (D) *Z. mays*, and (E) *P. patens* according to the STRING dataset. Figure S5. Protein interaction networks of CIN Class II TCP proteins in (A) *A. thaliana*, (B) *G. max*, (C) *O. sativa*, (D) *Z. mays*, and (E) *P. patens* according to the STRING dataset.
