# Supplementary material for: Evolutionary and Comparative Expression Analyses of TCP Transcription Factor Gene Family in Land Plants
Source: Int J Mol Sci. 2019 Jul 23;20(14):3591. doi: 10.3390/ijms20143591 (PMC6679135; doi:10.3390/ijms20143591)
Supplement: Supplementary file 1 [file ijms-20-03591-s001.zip › Supplementary Materials/Figure S1.pdf]

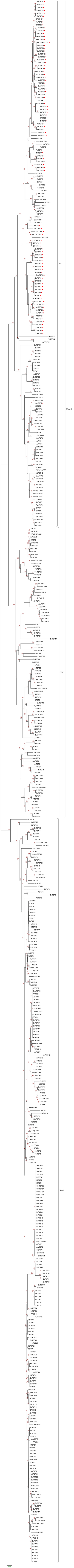
CIN
Class II
CYC
Class I

Figure S1. Phylogenetic tree of the 535 TCP proteins used in the present study. The unrooted tree was inferred by the neighbor-joining (NJ) method after the alignment of the TCP domains of the 535 candidate land plant TCP proteins listed in Table S1. Bootstrap values are indicated as percentages along the branches in the NJ analysis. The TCP proteins are clustered into two major classes (Class I and Class II). Class II was further divided into CIN and CYC clades. The red circle in the NJ tree represent the genes that were targeted by miR319.
