# Supplementary material for: Evolutionary and Comparative Expression Analyses of TCP Transcription Factor Gene Family in Land Plants
Source: Int J Mol Sci. 2019 Jul 23;20(14):3591. doi: 10.3390/ijms20143591 (PMC6679135; doi:10.3390/ijms20143591)
Supplement: Supplementary file 1 [file ijms-20-03591-s001.zip › Supplementary Materials/Figure S2.pdf]

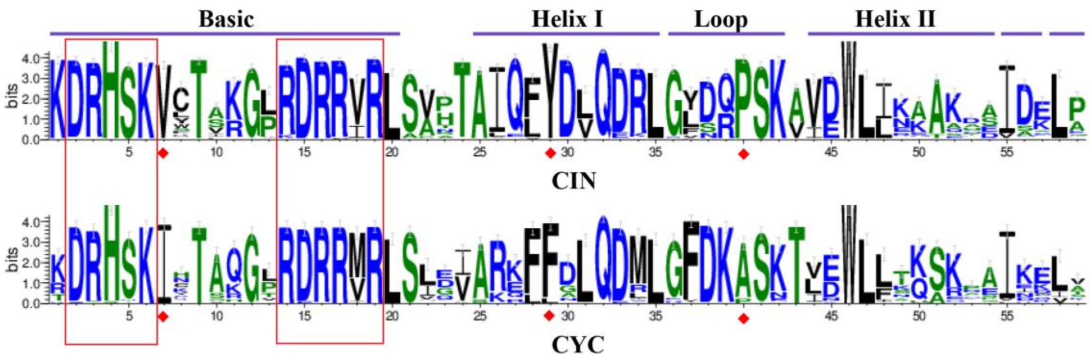

Figure S2. Sequence logos of the TCP domains in Class II TCP proteins. The bit score indicates the information content for each position in the sequence. Red rhomb indicates the conserved residues in CIN and CYC clades respectively. Red box was the conserved consensus sequence found in land plant TCP proteins.
