# Supplementary material for: Evolutionary and Comparative Expression Analyses of TCP Transcription Factor Gene Family in Land Plants
Source: Int J Mol Sci. 2019 Jul 23;20(14):3591. doi: 10.3390/ijms20143591 (PMC6679135; doi:10.3390/ijms20143591)
Supplement: Supplementary file 1 [file ijms-20-03591-s001.zip › Supplementary Materials/Figure S4.pdf]

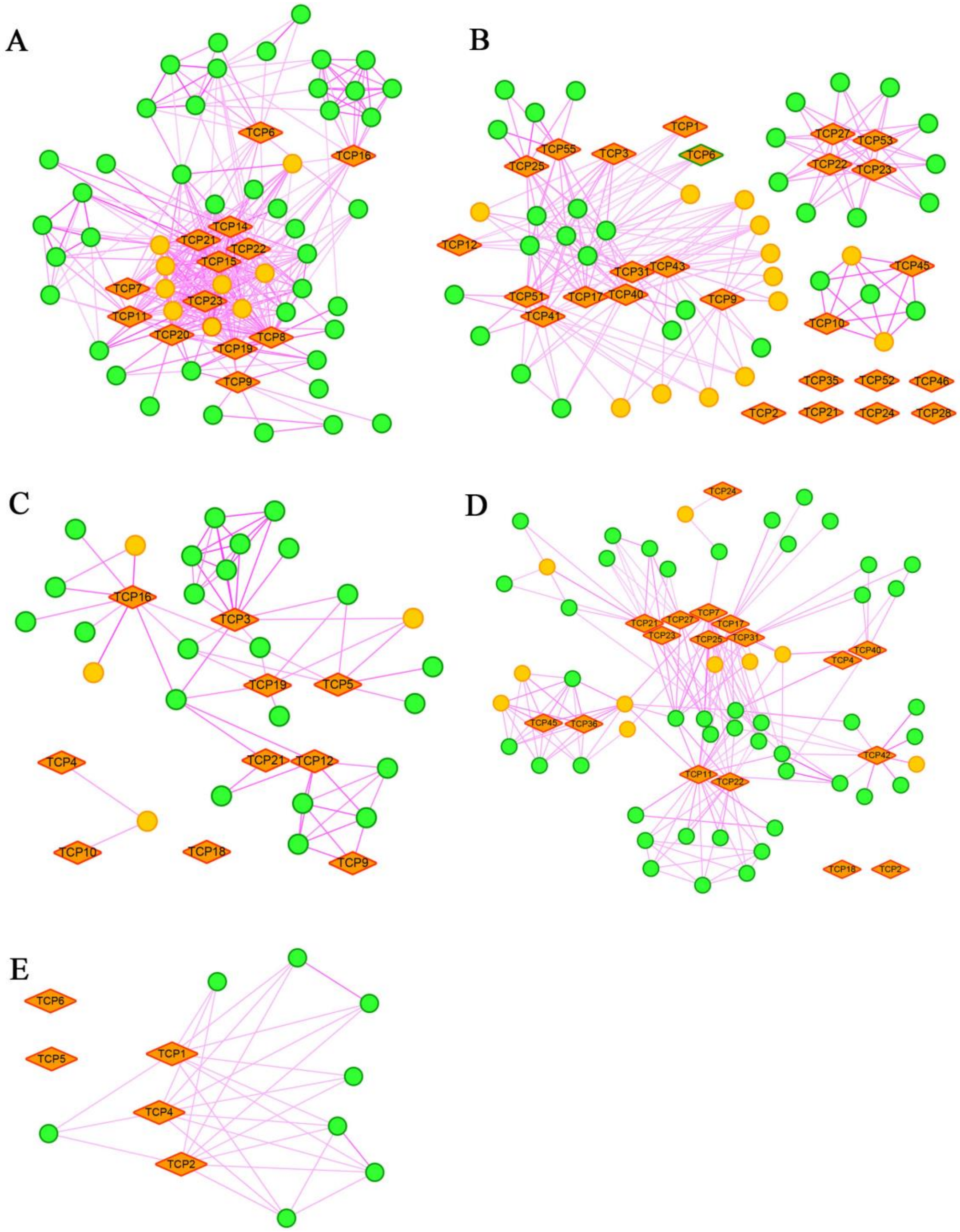

Figure S4. Protein interaction networks of Class I TCP proteins in (A) *A. thaliana*,(B) *G. max*, (C) *O. sativa*, (D) *Z. mays* and (E) *P. paten s* according to the STRING dataset. The Class I TCP proteins are in orange rhomb; the light orange circle represents other types of TCP proteins. Green circle represents non-TCP proteins .
