# Supplementary material for: Evolutionary and Comparative Expression Analyses of TCP Transcription Factor Gene Family in Land Plants
Source: Int J Mol Sci. 2019 Jul 23;20(14):3591. doi: 10.3390/ijms20143591 (PMC6679135; doi:10.3390/ijms20143591)
Supplement: Supplementary file 1 [file ijms-20-03591-s001.zip › Supplementary Materials/Figure S5.pdf]

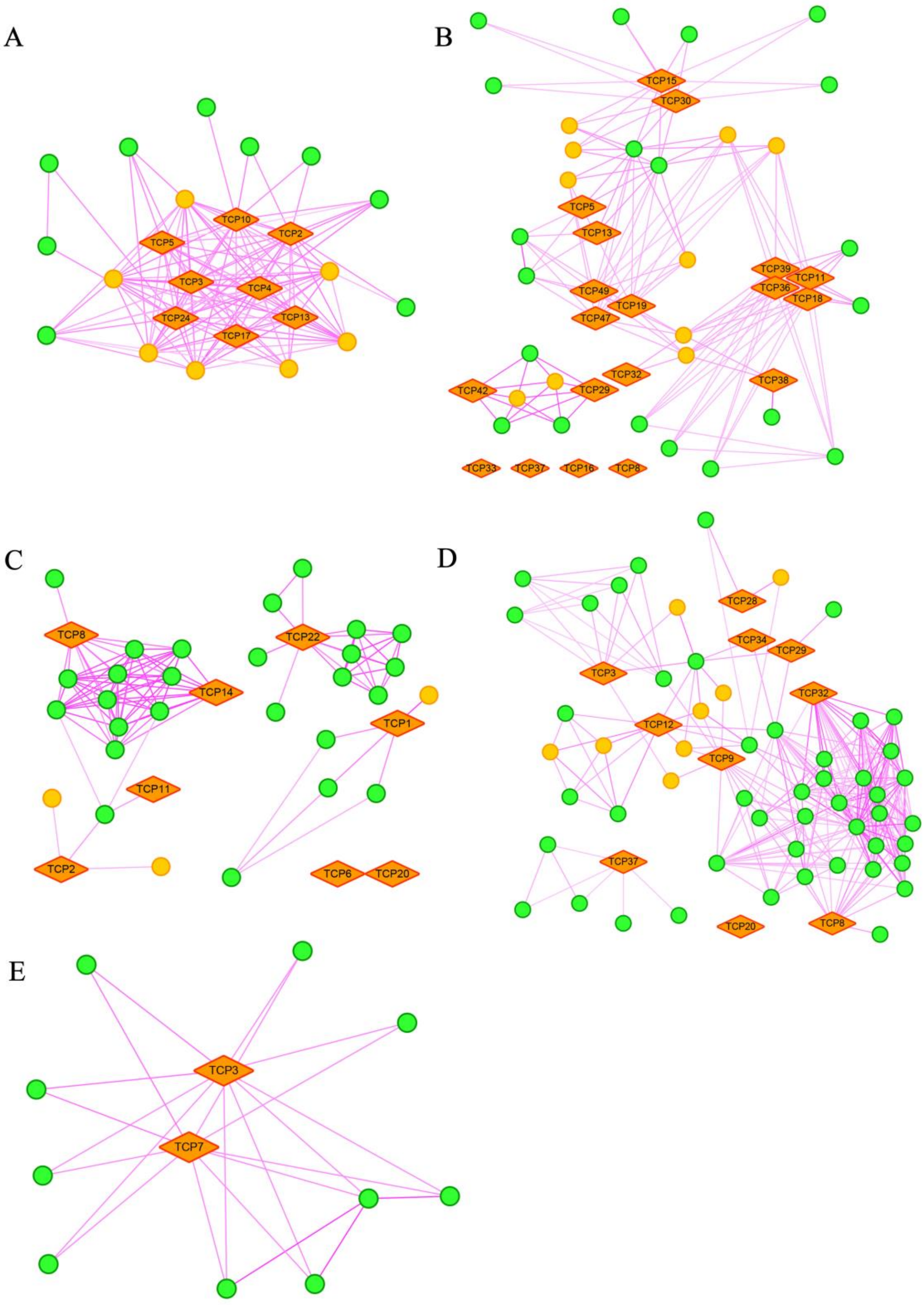

Figure S5. Protein interaction networks of ClN TCP proteins in (A) *A.thaliana*, (B) *G. max*, (C) *O. sativa*, (D) *Z. mays* and (E) *P. patens* according to the STRING datas et. The CIN TCP proteins are in orange rhomb; the light orange circle represents other types of TCP proteins. Green circle represents non-TCP proteins.
