# Supplementary material for: Evolutionary and Comparative Expression Analyses of TCP Transcription Factor Gene Family in Land Plants
Source: Int J Mol Sci. 2019 Jul 23;20(14):3591. doi: 10.3390/ijms20143591 (PMC6679135; doi:10.3390/ijms20143591)
Supplement: Supplementary file 1 [file Figure S3.pdf › Supplementary Materials/Figure S3.pdf]

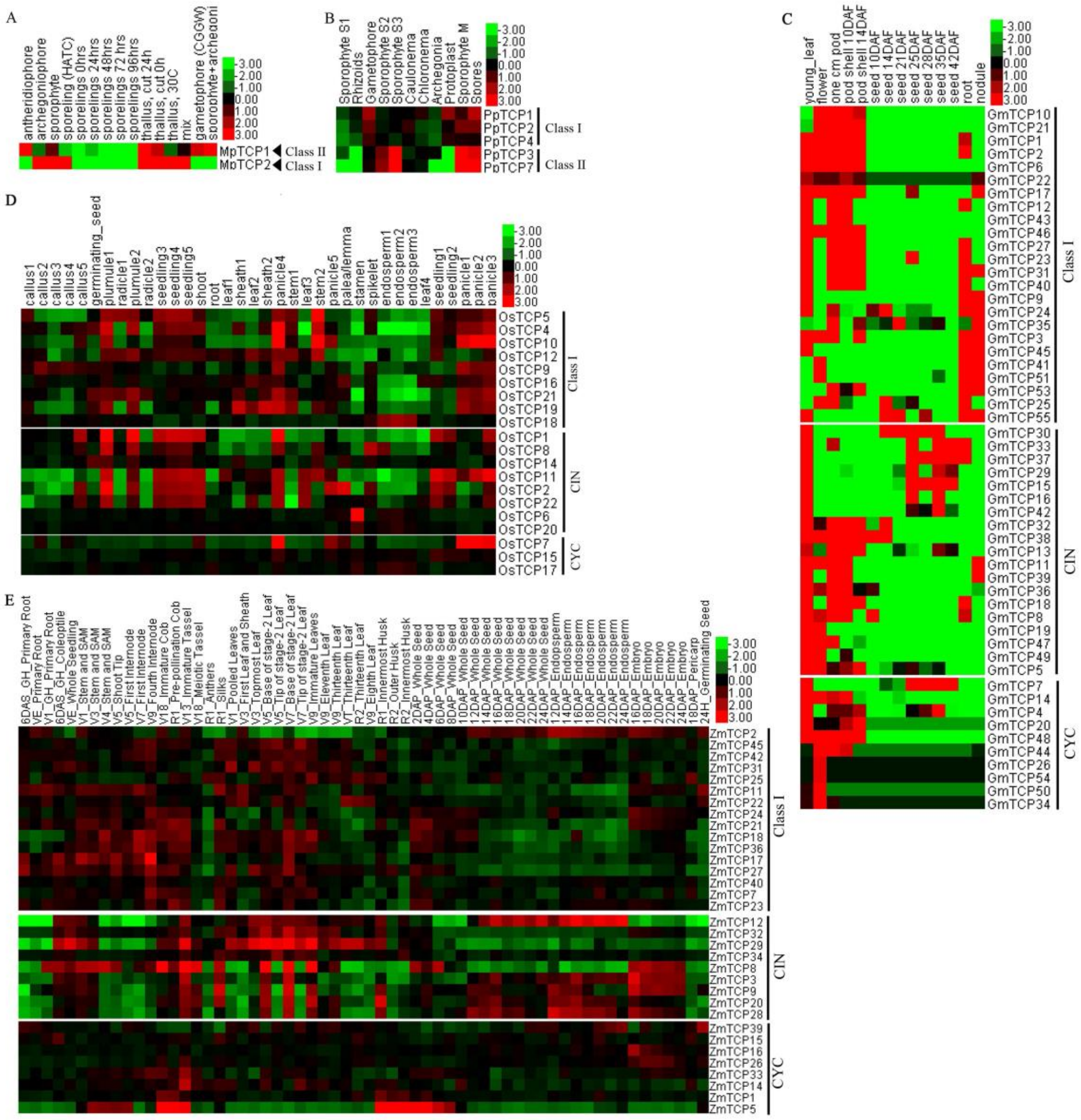

Figure S3. Expression profiles of TCP genes in *M. polymorpha*, *P. patens*, *G. max*, *Z. mays* and *O. sativa* across different developmental stages and organs. A: *M. polymorpha*; B: *P. patens*; C: *G. max*; D: *O. sativa*; E: *Z. mays*. The genes are listed on the right. The tissues used for expression analysis are indicated at the top of each column. The coloured bar represents $\log_2$ expression values. Red, green, and black indicate high, low, and medium expression levels, respectively.
